# A universal fluorescent sensing platform based on “signal switching induced by enzyme catalyzed substrate hydrolysis” for high-throughput discovery of xanthine oxidase inhibitors from traditional Chinese medicine

**DOI:** 10.3389/fphar.2026.1801989

**Published:** 2026-04-21

**Authors:** Guojing Liu, Kunhui Sun, Sitong Lai, Xiaoyi Liu, Yuanyuan Ge, Ping Wang, Bing Wang, Yuhong Li, Xie-An Yu

**Affiliations:** 1 Key Laboratory of Pharmacology of Traditional Chinese Medical Formulae, Ministry of Education, Tianjin University of Traditional Chinese Medicine, Tianjin, China; 2 NMPA Center for Innovation and Research in Regulatory Science, Shenzhen Institute for Drug Control, Shenzhen, China

**Keywords:** enzyme catalyzed substrate hydrolysis, fluorescent sensing platform, high-throughput screening, traditional Chinese medicine, xanthine oxidase inhibitor

## Abstract

**Introduction:**

Traditional Chinese medicine (TCM) serves as the treasure house for discovering lead compounds in drug development, demonstrating significant potential in the creation of anti-gout agents. However, given the complexity of TCM, conventional detection methods not only were susceptible to interference from metabolites but also suffered from low throughput, thereby hindering the efficient screening of inhibitors.

**Methods:**

This study developed a universal fluorescent sensing platform based on the principle of enzyme specific substrate recognition for the high-throughput screening of xanthine oxidase (XOD) inhibitors.

**Results:**

This approach offered two advances: (1) Interference-resistant detection: for screening in the complex system of TCM, its high emission wavelength (λem = 690 nm > 680 nm) and larger Stokes shift (Δλ = 148 nm) further enhanced anti-interference capability. (2) High-throughput screening capacity: by integrating HPLC-fully automatic partial fraction collector (HPLC-FC) and UHPLC-Q-TOF/MS, we established an “isolation-detection-identification” platform, screening and identifying four XOD inhibitors (Quercetin-3-sambubioside, Hyperoside, Isoquercitrin and Astragalin) from Nelumbinis Folium (NF) extract, with their combined inhibitory contribution accounting for 61.22% of the total inhibition activity.

**Discussion:**

Therefore, this strategy provided a powerful methodological tool for the high-throughput discovery of next-generation XOD inhibitors from TCM. And it offers a universal paradigm for the high-throughput discovery of bioactive metabolites from TCM targeting different diseases.

## 1 Introduction

Traditional Chinese medicine (Ge et al., 2023) (TCM) represents a vital resource for the discovery of lead compounds (Zhu et al., 2022; Liu et al., 2019), providing a rich material foundation for novel drug development in metabolic diseases (Ding et al., 2023). Taking hyperuricemia (Wang et al., 2025) and gout (Zhong et al., 2020) as the example, this disease have emerged as a significant public health issue affecting national health. The pathological basis (He et al., 2023; Calabuig et al., 2020) lies in the abnormal elevation of xanthine oxidase (Bui et al., 2016) (XOD) activity, leading to excessive uric acid production (Liu et al., 2022; Gan et al., 2024) and subsequently triggering gout attacks and associated cardiovascular risks (Bhattarai et al., 2024; Luo et al., 2022). Although current first-line clinical urate-lowering drugs (such as allopurinol (Zhang et al., 2024) and febuxostat (Zheng and Sun, 2022)) could effectively inhibit the activity of XOD (Patel and Thakkar, 2023), some patients experienced severe cutaneous allergic reactions (Liu et al., 2023), including life-threatening adverse events such as Stevens-Johnson syndrome (Wee et al., 2023), which necessitated treatment discontinuation. Although current first-line clinical urate-lowering drugs, such as allopurinol and febuxostat, could effectively inhibit the activity of XOD, their clinical utility is significantly hampered by specific and serious adverse reactions. Allopurinol is associated with allopurinol hypersensitivity syndrome (AHS), which can manifest as life-threatening Stevens-Johnson syndrome (SJS) and toxic epidermal necrolysis (TEN), particularly in genetically predisposed populations such as those carrying the HLA-B*5801 allele. This risk, which carries a mortality rate of up to 30%, mandates pretreatment genetic screening and limits its use. Febuxostat, while effective, carries relevant warnings, restricting its use in patients with cardiovascular comorbidities. Even the newer agent topiroxostat, though appearing safer in this regard, has limited global safety data. These clinical challenges highlight the urgent and unmet need for developing a new generation of efficient XOD inhibitors with improved safety profiles. Notably, numerous TCM have been demonstrated to possess significant XOD inhibitory activity, showing potential for lowering uric acid levels. Therefore, systematically screening TCM as a treasure trove of lead compounds to identify highly effective and low-toxicity XOD inhibitors not only aligns with its pharmacological basis but also offers a feasible pathway to overcome the limitations of current therapies. This highlighted the urgency for developing a new generation of efficient and safe inhibitors. Notably, numerous TCM had been demonstrated to possess significant XOD inhibitory activity, showing potential for lowering uric acid levels (Han et al., 2020; Tang et al., 2023). Therefore, systematically screening TCM as a treasure of lead compounds to identify highly effective and low-toxicity XOD inhibitors not only aligns with its pharmacological basis but also offers a feasible pathway to overcome the limitations of current therapies.

While TCM exhibited fine potential for lowering uric acid, its inherent complexity presented significant challenges in the efficient screening of high-potency inhibitors. On one hand, TCM contained a diverse array of compounds, including flavonoids, flavonoid-like substances, alkaloids and so on (Yan et al., 2022), whose absorption wavelengths predominantly fell within the near ultraviolet region. On the other hand, polyphenols and flavonoids (e.g., baicalein, quercetin), which were abundant in TCM extracts and possessed strong antioxidant properties, were prone to cause false-negative interference in conventional detection assays. The root cause of the existing technological bottlenecks lay in the multiple limitations inherent to current XOD activity detection systems. The prevailing methods primarily relied on the detection of enzymatic reaction products, either uric acid (Gęgotek et al., 2018; Son et al., 2019) (UV absorption at 290 nm) or hydrogen peroxide (Ha et al., 2011) (peroxidase-coupled assays). However, these approaches faced substantial interference when applied to TCM matrices: Ultraviolet-visible (UV-Vis) spectrophotometry detection was susceptible to interference from matrix metabolites absorbing in the near ultraviolet region and suffered from insufficient sensitivity, whereas hydrogen peroxide-based detection was severely quenched by the antioxidant metabolites in TCM, leading to missed active screening process. Furthermore, most of these methods involved product-based detection, making them vulnerable to interference and unsuitable for high-throughput screening requirements. These technical barriers rendered traditional approaches unsuitable for screening complex systems such as TCM, compelling the development of detection technologies based on the specific recognition of XOD.

**SCHEME 1:**
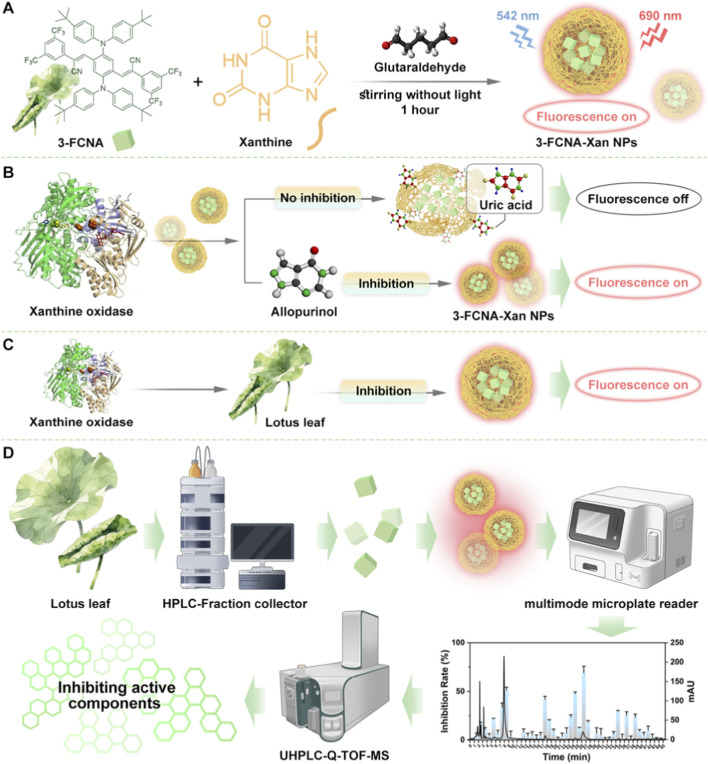
Schematic illustration of the fluorescent sensor 3-FCNA-Xan NPs for detection. **(A)** Synthesis of 3-FCNA-Xan NPs. **(B)** The schematic diagram of process of detecting XOD through 3-FCNA-Xan NPs. **(C)** Exploring the inhibitory effect of NF on XOD using 3-FCNA-Xan NPs. **(D)** Screening anti-XOD active metabolites via 3-FCNA-Xan NPs combined with UHPLC-Q-TOF/MS.

Currently, fluorescence-based methods are attracting attention due to their rapid response, operational simplicity, high sensitivity and compatibility with high-throughput screening. Recent advances continue to underscore this potential, with the development of novel platforms, such as Yahya S. Alqahtani et al. developed a BN@CDs probe based on the “ON-OFF-ON” fluorescence switching effect, which enabled the tandem fluorescence sensing of Al^3+^ and F^−^. Li et al. fabricated an AIE NP sensor with substrate-specific recognition capability based on the “ON-OFF-ON” fluorescence switching effect, which enabled the fluorescence detection of thrombin. Han et al. fabricated the TPE-S-TLG sensor by integrating the TPE-Ph-In probe with S-TLG, a SARS-CoV-2-specific substrate, based on the “ON-OFF-ON” fluorescence switching effect, and realized the fluorescence detection of SARS-CoV-2 (Alqahtani et al., 2023). Herein, we constructed a novel fluorescence sensor based on a strategy of “signal switching induced by enzyme catalyzed substrate hydrolysis”, which enabling the high-throughput screening (Chen et al., 2024) of XOD inhibitors from TCM. Specifically, nanoparticles (NPs) were formed using glutaraldehyde as the cross-linking agent, which reacted with the amino groups of xanthine (Xan). Upon encapsulation within these nanoparticles, 3-FCNA underwent aggregation, leading to a pronounced fluorescence signal. Upon XOD introduction, Xan undergoed specific enzymatic hydrolysis (Wu et al., 2018; Aladdin et al., 2020), triggering nanoparticle disassembly and consequent fluorescence quenching, establishing an negative correlation between enzymatic activity and signal output (R^2^ = 0.99, LOD = 0.27 U/L). XOD inhibitors attenuated fluorescence reduction by impeding substrate hydrolysis. Applied to Nelumbinis Folium (NF) extract, this sensor integrated HPLC-fully automatic partial fraction collector (HPLC-FC) and UHPLC-Q-TOF/MS to isolate and identify XOD inhibitory metabolites (Scheme 1). Critically, this sensor exhibited two attributes: (1) Interference-resistant detection: By monitoring substrate consumption rather than product formation, the 3-FCNA-Xan NPs effectively circumvented interference from endogenous enzymes and abundant antioxidant metabolites commonly present in TCM extracts. This anti-interference capability was further enhanced by the sensor’s larger Stokes shift (Δλ = 148 nm) and long emission wavelength (>680 nm), making it highly robust for applications in complex matrices. (2) High-throughput screening capability: By coupling the sensor with HPLC-FC and UHPLC-Q-TOF/MS, this work successfully screened and identified four XOD-inhibitory (Quercetin-3-sambubioside, Hyperoside, Isoquercitrin and Astragalin) fractions from the NF extract (Yu et al., 2025; Masuoka et al., 2006). Notably, these four metabolites collectively contributed 61.22% of the total XOD inhibitory activity of the extract, demonstrating the efficacy and high-throughput capacity of our approach. These results validate the proposed sensing platform as a robust tool for interference-resistant and high-throughput screening of XOD inhibitors from complex TCM matrices. Therefore, the developed strategy provided both a stable tool for XOD assay and an effective platform for inhibitor discovery from natural products. Its general applicability established a valuable paradigm for future lead compound identification against various targets and for navigating the chemical complexity of TCM.

## 2 Experimental

### 2.1 Reagents and materials

The 3-FCNA (Ruixi Biotechnology Co., Ltd., Xi’an, China), Xanthine (HY-W017389, MCE, China) and glutaraldehyde (Aladdin Chemistry Co., Shanghai, China) were purchased for preparation of the sensor to monitor of xanthine oxidase activity and screen natural inhibitor. Furthermore, xanthine oxidase was acquired from Shanghai Haoran Biotechnology Co., Ltd. (Shanghai, China) and Nelumbinis Folium (*Nelumbo nucifera* Gaertn; Nelumbonaceae; Leaf; Lot: 23061210; Production date: June 12, 2023; Storage conditions: room temperature) as a sample were obtained from Jiangxi Pengshi Guoyao Tang Pieces Co., Ltd. (Jiangxi, China). And allopurinol (HY-B0219, MCE, China) was used as a positive control. Acetonitrile (chromatographic grade for Liquid chromatography, mass spectrometry grade for Mass spectrometry analysis) and methanol (analytical pure for extraction) were obtained from Merck GmbH (Darmstadt, Germany), and tetrahydrofuran was supplied by Beijing Bailingwei Technology Co. (Beijing, China). The experimental water was purified using a Millipore water purification system (Millipore, Bedford, United States).

### 2.2 Instruments

The morphology of 3-FCNA-Xan NPs was observed by transmission electron microscopy (TEM, Talos F200X, Thermo Fisher Scientific, Waltham, MA, United States). Dynamic light scattering (DLS) and zeta potential were measured using a Malvern Instrument Zetasizer Nano 90 (Malver Instruments, Malvern, United Kingdom). Fourier transform infrared spectroscopy (FTIR) were recorded with a Bruker Company Vertex 70v model with Ram module II spectrometer (Bruker, Germany). Fluorescence data were obtained using a Varioskan Flash multimode microplate reader (Thermo Scientific, Vantaa, Finland). UV-Vis spectroscopy was conducted using a Shimadzu UV-2600 spectrophotometer (Japan). The sample to be tested was incubated at 37 °C in a KB53 biochemical incubator (Shanghai Truelab Laboratory Equipment Co., Ltd., Shanghai, China). High performance liquid chromatography (HPLC) analysis of NF extract was performed using the Shimadzu LC-20A High-Performance Liquid Chromatography (Japan). Subsequently, the NF fractions were collected using a DBS-100 fully automatic partial collector (Jingyi Instrument Equipment Co., Ltd., Beijing, China) and dried using an N-EVAP 111 nitrogen evaporator (Organomation, United States) to obtain samples of various NF fractions. The qualitative analysis of NF extract via UHPLC-Q-TOF/MS (AB Sciex, United States) were processed.

### 2.3 Synthesis of the 3-FCNA-Xan NPs

3-FCNA-Xan NPs were synthesized by a cross-linking method (Li et al., 2023; Han et al., 2024; Du et al., 2022). The synthesis conditions for 3-FCNA-Xan NPs, encompassing factors such as substrate concentration, stirring duration, stirring rate, and the proportion of glutaraldehyde, were optimized using the controlled variable method. Specifically, 3-FCNA-Xan NPs were successfully synthesized through the aggregation of 3-FCNA and Xan crosslinked with glutaraldehyde. In detail, 100 μL of THF solution containing 3-FCNA (10 mg/mL) was added to a vial containing 900 μL of Xan (1 mM) solution at a rate of 25 μL/min using a 100 μL pipette, followed by the addition of 9 μL of 25% glutaraldehyde. The mixture was then stirred at 300 rpm on a magnetic stirrer at room temperature, under sealed and light-avoiding conditions, for 60 min. After the reaction, the mixture was allowed to stand in the dark for 12 h to allow the THF to evaporate. The volume was then adjusted back to the original, yielding the 3-FCNA-Xan NPs. The synthesized 3-FCNA-Xan NPs were stored in a refrigerator at 4 °C and protected from light for subsequent use.

### 2.4 Characterization of the 3-FCNA-Xan NPs

The characterization of 3-FCNA-Xan NPs included morphology, hydrated particle size, zeta potential, fourier transform infrared spectroscopy, UV-vis spectroscopy and fluorescence spectroscopy. Among them, the morphology of 3-FCNA-Xan NPs was visually observed through transmission electron microscopy, DLS and zeta potential measurements were performed using Malvern Instrument Zetasizer Nano 90. Furthermore, fourier transform infrared spectroscopy was used to perform FTIR spectroscopy to confirm the accomplished preparation of 3-FCNA-Xan NPs. In addition, UV data and fluorescence data were measured using UV-2600 spectrophotometer and Varioskan Flash multimode microplate reader to further determine the signal changes induced by aggregation depolymerization of 3-FCNA-Xan NPs, reflecting the completed preparation of 3-FCNA-Xan NPs.

### 2.5 Detection of XOD by the 3-FCNA-Xan NPs

The activity of XOD was measured using the synthesized 3-FCNA-Xan NPs. First, PBS buffer was added to the wells of a black 96-well plate and incubated for 5 min at 37 °C for pre-warming to provide the enzyme with a suitable temperature. Subsequently, XOD was added, followed by another 5 min incubation. Then, 50 µL of the 3-FCNA-Xan NPs solution was added. The final reaction volume was adjusted to 100 µL with PBS buffer. Fluorescence was measured immediately using a multimode microplate reader (λex/λem = 542/690 nm). Allopurinol, a known XOD inhibitor, was used as a positive control.

Using the above detection program as a template, different concentrations of XOD (0.5, 5, 50, 100, 200, 300, 400, 500 and 600 U/L) were added to 96-well black plate, shaken solwly, and incubated for 5 min. Then, 50 μL of 3-FCNA-Xan NPs solution was added and supplemented with PBS buffer to 100 μL. Fluorescence detection was performed in a multimode microplate reader (λex/λem = 542/690 nm) to determine the enzyme activity curve.

### 2.6 Screening anti-XOD metabolites from NF extract by the 3-FCNA-Xan NPs

To detect the inhibitory effect of NF extract on XOD, the prepared 3-FCNA-Xan NPs were used to monitor the inhibitory effect of NF extract on XOD activity. Based on the protocol in Section 2.5, incubation with XOD was carried out for 5 min before the addition of 10 μL of NF extract, after which the incubation continued for an additional 10 min. Subsequently, 50 μL of 3-FCNA-Xan NPs were added and supplemented with PBS buffer to 100 μL, and fluorescence detection was performed in a multimode microplate reader (λex/λem = 542/690 nm).

Then, the metabolites in NF were separated using HPLC. Firstly, many experimental conditions such as mobile phase compositions, detection wavelength, flow rates, column temperature and gradient elution methods were optimized to construct the HPLC fingerprint of NF extract. Then, at intervals of 1 min, the fractions in the NF extract were collected using a HPLC-FC. The enriched fractions were dried with nitrogen and then dissolved in an extraction solvent to obtain a NF fraction sample solution. 3-FCNA-Xan NPs were employed to evaluate the anti-XOD activity of each fraction using the same detection method described above. Fractions exhibiting notable anti-XOD activity in NF were qualitatively analyzed by UHPLC-Q-TOF/MS to identify their structures. Finally, these active fractions were compared with standard samples to determine the specific metabolites in NF responsible for XOD inhibition. These active metabolites were further validated through 3-FCNA-Xan NPs to obtain their half-maximal inhibitory concentration (IC_50_). Choose the two concentrations closest to an enzyme inhibition rate of 50% and their corresponding enzyme inhibition rates for calculation. One of these two concentrations must be greater than 50%, and the other must be less than 50%. The concentration below 50% is defined as D_1_, with an inhibition rate of P_1_; the concentration above 50% is defined as D2, with an inhibition rate of P_2_. The formula is calculated as IC_50_ = D_1_ + [(D_2_ - D_1_)(50 - P_1_)]/(P_2_ - P_1_). In addition, considering practical conditions and the ease of obtaining standard substances, the HPLC method was validated for four compounds in terms of linearity, precision, stability, repeatability and matrix recovery. Finally, based on the composition of the NF extract, a mixed reference standard was prepared to contain each compound at the same concentration as present in the extract. The anti-XOD activity of this reconstituted mixture was then compared with that of the original whole extract.

## 3 Results and discussion

### 3.1 AIE properties of 3-FCNA

**FIGURE 1:**
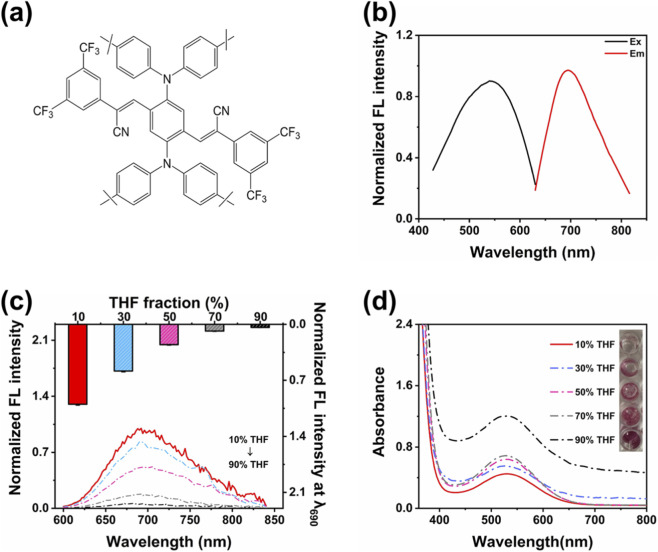
AIE characteristics of 3-FCNA. **(a)** The structure of 3-FCNA. **(b)** Normalized fluorescence excitation and emission spectra of 3-FCNA in 10% THF-H_2_O mixed solvent. **(c)** Normalized fluorescence emission spectra of 3-FCNA in THF-H_2_O mixed solutions with different volume fractions. **(d)** UV-Vis absorption spectroscopy of 3-FCNA in THF-H_2_O mixed solutions with different volume fractions. Data are represented as mean ± standard deviation (SD). All error bars represent standard deviations based on three parallel measurements.

The structure of the 3-FCNA was demonstrated in Figure 1a. The compound was designed with structures consisting of benzene ring conjugated skeleton, push-pull electron withdrawing groups, and nitrogen atom conjugated connection, which satisfied the “intramolecular rotation restriction aggregation state planarization luminescence enhancement” mechanism of AIE effect, thus exhibiting AIE effect (Lu et al., 2016). Specifically, the benzene ring in the compound was mainly connected by ethylene bonds, giving it a conjugated system that tends to flatten and a rigid skeleton, which helped to improve electronic transition efficiency; Secondly, the strong electron withdrawing properties of trifluoromethyl (CF_3_) and cyano (CN) groups led to a decrease in electron cloud density, forming a “push-pull electron” equilibrium with the electron donating properties of methyl (CH_3_). This not only stabilized the electronic structure of the conjugated system, but also regulated the intramolecular rotation and aggregation conformation through electronic effects; Finally, the conjugated chain involving nitrogen atoms further extended the electron transition path, enhances intramolecular electron coupling, and provided electronic structural support for improving the luminescence efficiency of aggregated states. The ^1^H-NMR spectrum of 3-FCNA was shown in Supplementary Figure S1. And the MSMS fragment of 3-FCNA was shown in Supplementary Figure S2. As shown in Figure 1b, the maximum excitation wavelength of 3-FCNA was 542 nm, and its emission wavelength was 690 nm. The larger Stokes shift (Niu et al., 2022) (Δλ = 148 nm) and high emission wavelength (>680 nm) further enhanced the detection anti-interference ability, making it suitable for detection applications in complex matrices. In addition, 3-FCNA was dissolved in V_(THF)_/V_(H2O)_ solutions at different ratios. As the volume ratio of THF in the mixed solution increased, the fluorescence intensity of 3-FCNA significantly decreased. It clearly aggregated with obvious fluorescence in 10% THF-H_2_O solution and exhibited favourable AIE effect (Figure 1c). Eventually, further investigation was conducted on the UV-Vis absorption spectroscopy of 3-FCNA in THF-H_2_O mixed solutions with different volume fractions at the same concentration. As the volume ratio of THF in the mixed solution increased, it was found that the UV absorption of 3-FCNA also continued to increase, indicating that 3-FCNA could dissolve well in THF solution (Figure 1d). Once again, it has been demonstrated that 3-FCNA exhibited significant aggregation in a 10% THF-H_2_O solution. Based on the above results, the fine AIE performance of 3-FCNA was further demonstrated, and the THF:H_2_O = 1:9 ratio was identified as optimal for use in subsequent sensor fabrication.

### 3.2 Synthesis and characterization of 3-FCNA-Xan NPs

**FIGURE 2:**
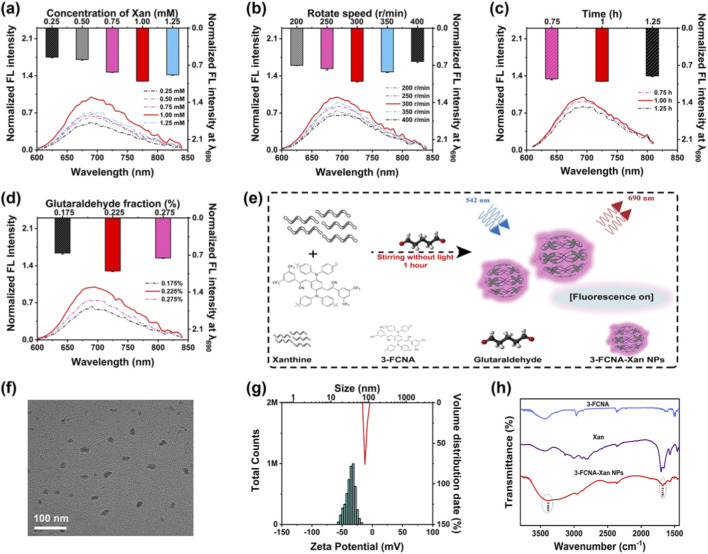
Synthesis and characterization of 3-FCNA-Xan NPs. **(a)** Normalized fluorescence spectra of 3-FCNA-Xan NPs synthesized at different substrate concentration. **(b)** Normalized fluorescence spectra of 3-FCNA-Xan NPs synthesized at different rotation speed. **(c)** Normalized fluorescence spectra of 3-FCNA-Xan NPs synthesized at different reaction time. **(d)** Normalized fluorescence spectra of 3-FCNA-Xan NPs synthesized at different the proportion of glutaraldehyde. **(e)** The schematic diagram of synthesis process of 3-FCNA-Xan NPs. **(f)** TEM image of 3-FCNA-Xan NPs. **(g)** Zeta potential and hydrodynamic size of 3-FCNA-Xan NPs (1 M = 1 × 10^6^). **(h)** FTIR spectrum of 3-FCNA, Xan and 3-FCNA-Xan NPs. Data are represented as mean ± SD. All error bars represent standard deviations based on three parallel measurements.

Based on the strategy of “enzyme-triggered-substrate nanoparticle disassembly fluorescence modulation”, 3-FCNA-Xan NPs were constructed through crosslinking with glutaraldehyde. Under the action of the magnetic whisk, nanoparticles were formed by crosslinking glutaraldehyde with xanthine, and the probe 3-FCNA was wrapped to form 3-FCNA-Xan NPs. After dissolving 3-FCNA in THF, the synthesis conditions of 3-FCNA-Xan NPs were optimized by controlling variables, including substrate concentration, stirring rate, stirring duration and the proportion of glutaraldehyde. Specifically, the results in Figure 2a indicated that as the substrate concentration increased from 0.25 mM to 1 mM, the fluorescence intensity of 3-FCNA-Xan NPs significantly increased. However, as the substrate concentration increased further, the fluorescence intensity gradually decreased. Figure 2b showed the exploration of stirring speed during the preparation of 3-FCNA-Xan NPs, and it was found that the fluorescence of 3-FCNA-Xan NPs was highest at 300 rpm. Figures 2c,d showed the investigation of reaction time and the proportion of glutaraldehyde in the preparation process of 3-FCNA-Xan NPs. The results manifested that the fluorescence effect of 3-FCNA-Xan NPs was optimal when the reaction time was 1 h and the proportion of glutaraldehyde was 0.225%. Therefore, the optimal synthesis conditions for 3-FCNA-Xan NPs were ultimately determined as follows (Figure 2e): substrate concentration of 1 mM, stirring rate of 300 rpm, stirring duration of 1 h and the proportion of glutaraldehyde of 0.225%.

Subsequently, the successfully prepared 3-FCNA-Xan NPs were characterized using TEM, DLS and FTIR spectroscopy. Among them, the TEM image revealed uniform particle-like distribution of 3-FCNA-Xan NPs (Figure 2f), which intuitively demonstrated the successful preparation of the nanoparticles. Secondly, the average hydrated particle size of 3-FCNA-Xan NPs and 3-FCNA were measured to be 83.02 nm (Figure 2g) and 59.07 nm (Supplementary Figures S3), while the zeta potentials of 3-FCNA-Xan NPs, 3-FCNA and Xan were determined as −36.22 mV, −26.53 mV and −4.28 mV (Figure 2g, Supplementary Figure S4, S5). The increase in particle size and absolute zeta potential value of 3-FCNA-Xan NPs compared to other groups further confirmed the successful fabrication of the sensor and demonstrated its stable colloidal state. The nanoparticles exhibited stable hydrodynamic diameter, zeta potential, and fluorescence intensity over 7 days (Supplementary Figure S6). Meanwhile, the infrared spectrum showed an enhanced and broadened peak at 3386.2 cm^−1^, which might arise from the superposition of the C=N stretching vibration in the schiff base and the -N-H stretching vibration of residual -N-H groups. The peak at 1677.5 cm^−1^ decreased, corresponding to the weakened C=O stretching vibration in xanthine due to deprotonation or cross-linking reactions, further confirming the successful preparation of 3-FCNA-Xan NPs (Figure 2H). In order to evaluate the stability of the sensor, the fluorescence intensity of 3-FCNA-Xan NPs was measured continuously for 7 days at the same time period. The results showed minimal variation in fluorescence intensity over the week (Supplementary Figure S6), indicating excellent fluorescence stability and suitability for further screening applications. In addition, the fluorescence response of 3-FCNA-Xan NPs under different conditions was investigated. The results demonstrated that 3-FCNA-Xan NPs only responded to XOD, resulting in a decrease in fluorescence intensity, while other groups had negligible effects on the fluorescence of the sensor, consistent with pure 3-FCNA-Xan NPs (Supplementary Figure S7), indicating that 3-FCNA-Xan NPs possessed high exclusive responsiveness to XOD.

### 3.3 Detection of XOD based on the 3-FCNA-Xan NPs

**FIGURE 3:**
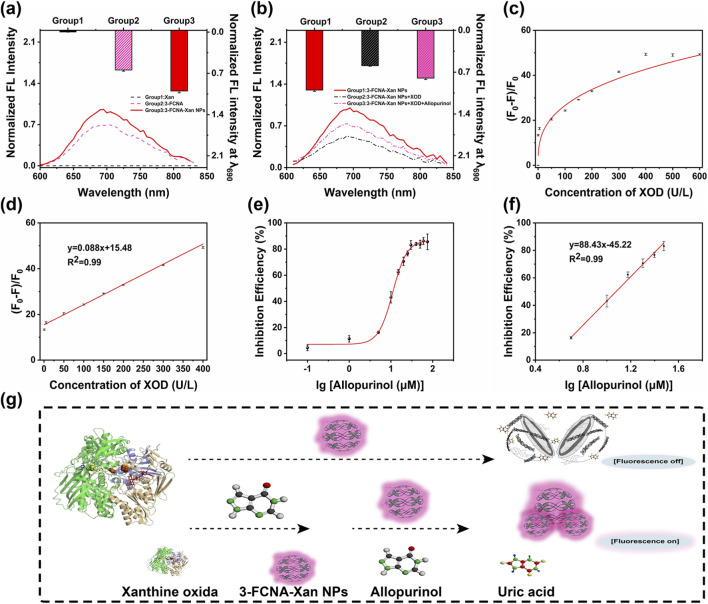
The specific enzyme responsiveness of 3-FCNA-Xan NPs. **(a)** Normalized fluorescence spectra of different solutions such as Xan, 3-FCNA and 3-FCNA-Xan NPs. **(b)** Normalized fluorescence spectra of 3-FCNA-Xan NPs, 3-FCNA-Xan NPs + XOD and 3-FCNA-Xan NPs + XOD + Allopurinol. **(c)** The relationship between different concentrations of XOD and the fluorescence quenching efficiency of 3-FCNA-Xan NPs. **(d)** The linear relationship between the fluorescence quenching efficiency of 3-FCNA-Xan NPs and the concentration of XOD (0.5–400 U/L). **(e)** The inhibitory effect of different concentrations of allopurinol on XOD. **(f)** The linear relationship between the inhibition rate of XOD by allopurinol and the concentration of allopurinol (5–30 μM). **(g)** The schematic diagram of process of detecting XOD through 3-FCNA-Xan NPs. Data are represented as mean ± SD. All error bars represent standard deviations based on three parallel measurements.

As shown in Figure 3a, at 690 nm, the substrate Xan alone did not exhibit fluorescence, while the synthesized 3-FCNA-Xan NPs demonstrated higher fluorescence intensity than the probe 3-FCNA, further confirming the successful preparation of 3-FCNA-Xan NPs. Subsequently, the reaction time between 3-FCNA-Xan NPs and XOD was investigated (Supplementary Figure S8), revealing that within 5 min, the fluorescence intensity of 3-FCNA-Xan NPs decreased with prolonged incubation time. However, beyond 5 min, its fluorescence intensity showed little change. Therefore, considering the adequate and fast reaction time of 3-FCNA-Xan NPs with XOD, 5 min reaction time was selected for the next experimental work. In addition, the optimal incubation time for XOD and inhibitors were explored separately. As shown in Supplementary Figure S9 5 min was selected as the optimal pre-incubation time to ensure consistent baseline enzyme activity for subsequent inhibition studies. The results (Supplementary Figure S10) showed that the fluorescence intensity recovered (indicating inhibition of XOD activity) with increasing time until it reached a plateau at approximately 10 min. No significant change in fluorescence was observed beyond this point, confirming that 10 min is sufficient for the interaction between XOD and the inhibitor to reach equilibrium.

On the other hand, the responsiveness of 3-FCNA-Xan NPs to XOD was further validated. Given that XOD specifically hydrolyzes Xan, this enzymatic reaction could break down the 3-FCNA-Xan NPs, leading to a decrease in the fluorescence intensity of 3-FCNA-Xan NPs. When XOD inhibitors were presented, they inhibited the activity of XOD, resulting in a weakened reduction in the fluorescence intensity of 3-FCNA-Xan NPs (Figure 3b). This further confirmed the feasibility of using 3-FCNA-Xan NPs to detect XOD. Additionally, UV-Vis spectrophotometry was employed to detect the reaction products of XOD and Xan. After incubating Xan with XOD for 5 min, the UV-Vis spectrum showed an absorption peak of uric acid at 290 nm (Supplementary Figure S11). And under the same conditions, 3-FCNA-Xan NPs showed the same absorption peak at 290 nm after co-incubation with XOD. Fortunately, the positive control group showed that after adding allopurinol, the absorption peak at 290 nm in the UV-Vis spectrum decreased, indicating that allopurinol could inhibit the activity of XOD, thereby reducing the production of uric acid (Supplementary Figure S12). UV-Vis spectroscopy showed that when XOD inhibitors were presented, the amount of uric acid produced also decreases. From this perspective, it could be better demonstrated that 3-FCNA-Xan NPs could be used to detect the activity of XOD.

Afterward, the relationship between XOD concentration and the fluorescence intensity of 3-FCNA-Xan NPs was studied. The results showed that with the increase of XOD concentration, the fluorescence intensity of 3-FCNA-Xan NPs gradually decreased, and its fluorescence quenching efficiency gradually increased, showing a linear relationship in the range of 0.5–400 U/L (R^2^ = 0.99), with a detection limit of 0.27 U/L (Figures 3c,d). It was found that the fluorescence intensity remained constant at XOD concentrations above 400 U/L. Therefore, 400 U/L was selected as the concentration for screening anti-XOD active substances in the next experiments. Similarly, the allopurinol was tested using 3-FCNA-Xan NPs, with an IC_50_ value of 11.80 μM (Figures 3e,f). In addition, we spiked three different concentrations of Allopurinol into the reaction matrix and calculated the recovery rates. The recovery rates ranged from 98.22% to 103.24% (Supplementary Table S1), indicating that there is no significant matrix suppression or enhancement under our experimental conditions. Hence, Figure 3g illustrated the process of detecting XOD using 3-FCNA-Xan NPs. XOD could specifically hydrolyze Xan, leading to the rupture of 3-FCNA-Xan NPs and causing the fluorescence of 3-FCNA-Xan NPs to appear “off”. When XOD inhibitors were presented, they inhibited the activity of XOD, affecting the efficiency of the reaction between XOD and Xan. As a result, the 3-FCNA-Xan NPs still maintained fluorescence and appeared “on”.

### 3.4 Screening of anti-XOD active substances using 3-FCNA-Xan NPs combined with UHPLC-Q-TOF/MS and validation of anti-XOD activity of NF extract and mixed standard

**FIGURE 4:**
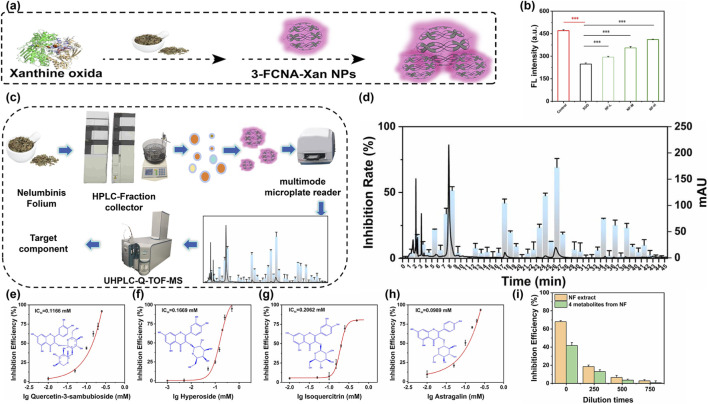
The inhibitory effect of NF extract on XOD. **(a)** Exploring the inhibitory effect of NF on XOD using 3-FCNA-Xan NPs. **(b)** Fluorescence intensity of 3-FCNA-Xan NPs under different doses of NF extract. **(c)** The chromatogram of NF extract and the inhibition rate of each component of NF extract on XOD. **(d)** Screening anti-XOD active substances using 3-FCNA-Xan NPs combined with UHPLC-Q-TOF/MS. **(e)** The inhibitory effect of quercetin-3-sambubioside at different concentrations on XOD. **(f)** The inhibitory effect of different concentrations of hyperoside on XOD. **(g)** The inhibitory effect of different concentrations of isoquercitrin on XOD. **(h)** The inhibitory effect of different concentrations of astragalin on XOD. **(i)** The inhibitory effect of NF extract and its four corresponding mixed standard solutions (Quercetin-3-sambubioside, Hyperoside, Isoquercitrin and Astragalin) on XOD. Data are represented as mean ± SD. All error bars represent standard deviations based on three parallel measurements.

Firstly, 3-FCNA-Xan NPs were used to investigate the inhibitory effect of NF on XOD, as shown in Figure 4a. The results indicated that as the concentration of NF extract increased, the decline extent in fluorescence intensity of 3-FCNA-Xan NPs was gradually attenuated, implying the inhibitory effect of NF on XOD (Figure 4b) and the presence of natural XOD inhibitory metabolites in NF. In addition, the NF extract alone did not produce fluorescence at an emission wavelength of 690 nm, and the fluorescence intensity of 3-FCNA-Xan NPs was almost unaffected after co incubation with 3-FCNA-Xan NPs (Supplementary Figure S13). This indicated that NF extract could not interfere with the fluorescence of 3-FCNA-Xan NPs. Secondly, by systematically examining and optimizing various HPLC conditions, the fingerprint spectrum of NF extract was established. Specifically, key parameters such as the composition of the mobile phase (Supplementary Figure S14), detection wavelength (Supplementary Figure S15), flow rate (Supplementary Figure S16), column temperature (Supplementary Figure S17) and elution gradient were comprehensively screened and optimized. On this basis, combined with the requirements of chromatographic peak separation and baseline stability, the extraction solvent system of NF were further optimized (Supplementary Figure S18) and finally a 50% methanol solution was determined as the initial extraction solvent. The HPLC conditions optimized using NanoChrom chromatography column (ChromCore C18, 5 μm, 4.6 mm × 250 mm) were as follows, water (phase A)-acetonitrile (phase B) was selected as the mobile phase, detection wavelength was 254 nm, flow rate was 1.0 mL/min, column temperature was 30 °C and elution gradient was shown in Supplementary Table S2. The extraction procedure for NF were as follows, accurately 0.5 g of NF powder, passed through a No.3 sieve, was weighed and transferred into a 50 mL volumetric flask. The flask was then filled to the mark with a 50% methanol solution and its initial weight was recorded. Next, the flask was subjected to ultrasonic extraction at room temperature for 1 h. After extraction, the flask was cooled to room temperature, reweighed and any solvent loss due to evaporation was compensated for by adding 50% methanol solution. After shaking the mixture well, it was pre-filtered through qualitative filter paper and then filtered through a 0.45 μm microporous membrane. And the filtrate was collected as NF extract. Then the extract was stored at 4 °C in the dark for future experiments.

Using HPLC-FC technology, the metabolites in the NF extract were collected step by step at 1 min intervals. After drying the collected fractions with nitrogen, the NF component sample solution was prepared by dissolving them in the initial extraction solvent. The inhibitory activity of XOD on various metabolites of NF extract was screened using 3-FCNA-Xan NPs and the inhibitory effect was shown in Figure 4c. To further verify the accuracy of component collection, solutions of metabolites with obvious anti-XOD activity (Fraction 18, 23, 25, 27, 35, 36 and 38) were re-injected for analysis (Supplementary Figures S19–S26). The chromatographic conditions such as mobile phase, detection wavelength, column temperature and flow rate were consistent with the original NF extract, and the consistency of the target peak was confirmed by comparing the retention time.

To clarify the structural characteristics of the anti-XOD active metabolites in NF, UHPLC-Q-TOF/MS was used for qualitative analysis and the screening process was shown in Figure 4d. By obtaining molecular fragment information of chemical metabolites through UHPLC-Q-TOF/MS and comparing with literature databases, six metabolites were successfully identified in negative ion mode (Supplementary Figures S27–S33). The determination of the chemical composition of NF was based on both the mass spectrometry characteristics of known metabolites in the reference literature and the molecular fragment information obtained by UHPLC-Q-TOF/MS (Supplementary Table S3). Taking into account the availability of standard samples and their practical value, four active metabolites were ultimately selected (Quercetin-3-sambubioside, Hyperoside, Isoquercitrin and Astragalin). To establish quality control standards for NF extract, methodological validation was conducted on the four metabolites mentioned above, including the following.

The calibration curve demonstrated good linearity within the established range (R^2^ > 0.999), with the linear response range detailed in Supplementary Table S4. Regarding precision, the intra-day relative standard deviation (RSD) was below 2.10%, while the inter-day RSD was less than 1.52% (Supplementary Table S5). In terms of stability, the RSD of the sample throughout the investigated period remained under 2.55% (Supplementary Table S5). And repeatability testing yielded an RSD below 1.58% (Supplementary Table S6), indicating excellent method reproducibility. For accuracy, the sample recovery rate ranges from 99.4% to 103.78%, with an RSD of ≤1.50% (Supplementary Table S7), meeting the requirements for quantitative analysis. The chromatograms for specificity (Supplementary Figure S34), along with data from linearity and response range, precision and stability, and repeatability, collectively demonstrate that the method exhibited excellent stability and reproducibility, making it suitable for the accurate determination of the four active metabolites in NF extract. The individual inhibition rates of these four standard metabolites were shown in Figures 4e–h, indicating that four metabolites had effective anti-XOD activity. According to the content of each compound in the NF extract, prepared a mixed standard with the same concentration and compared its anti-XOD activity with the NF extract. The results showed that these four metabolites achieved 61.22% anti-XOD activity of NF extract (Figure 4i). Meanwhile, the inhibitory effect of these four standard substances on XOD was similar to that of NF extract, and the inhibitory effect decreases with the increase of dilution ratio, indicating that these four active metabolites could represent 61.22% of the anti-XOD activity of NF extract and could be used for quality evaluation of NF extract. To further validate the reliability of the 3-FCNA-Xan NPs-based method, we conducted a cross-validation experiment using the conventional xanthine substrate assay. First, we established a standard curve correlating uric acid absorbance with XOD concentration using xanthine as the substrate (Supplementary Figure S35). As shown as Supplementary Figure S35, the absorbance at the maximum UV peak of uric acid exhibited a good linear relationship with XOD concentration in the range of 0.5–50 U/L, Y = 0.015X + 0.01798, R^2^ = 0.991. Subsequently, we applied the IC_50_ concentrations of compounds Quercetin-3-sambubioside, Hyperoside, Isoquercitrin and Astragalin previously determined by the 3-FCNA-Xan NPs-based method, to the xanthine substrate system and measured their inhibition rates at these concentrations. The results showed that the inhibition rates of all four compounds in this validation experiment were approximately 50%, ranging from 48% to 52% (Supplementary Figure S36). These findings demonstrate that the IC_50_ values obtained by our 3-FCNA-Xan NPs method are consistent with those expected from the conventional xanthine assay, further confirming the accuracy and reliability of the proposed method.

## 4 Conclusion

This study successfully developed a novel fluorescence sensor based on a strategy of “signal switching induced by enzyme catalyzed substrate hydrolysis”, which enabling the high-throughput screening of XOD inhibitors from TCM. By integrating HPLC-FC and UHPLC-Q-TOF/MS, we established an “isolation-detection-identification” platform, screening and identifying four XOD inhibitors (Quercetin-3-sambubioside, Hyperoside, Isoquercitrin and Astragalin) from NF extract, with their combined inhibitory contribution accounting for 61.22% of the total inhibition activity. Despite this successful identification, the four metabolites accounted for only 61.22% of the total inhibitory activity, leaving approximately 38.78% unexplained. This residual activity could be attributed to trace compounds below the sensor’s detection threshold, or synergistic interactions between the identified flavonoids and other metabolites. Future studies employing enrichment strategies and combinatorial index analysis could address these possibilities, enabling a more comprehensive profiling of bioactive compounds from complex matrices.

The four identified flavonoid glycosides were known metabolites of NF, and their aglycone skeletons (quercetin/kaempferol) had been extensively reported as XOD inhibitors. Compared to conventional screening methods, this fluorescence platform offered superior throughput and anti-interference capability. The sensor exhibited a low LOD for XOD inhibitors, ensuring that even relatively weak inhibitors could be reliably identified. More importantly, the high emission wavelength (690 nm) and large Stokes shift (148 nm) of the AIE probe effectively circumvented the autofluorescence commonly encountered in complex TCM matrices, thereby mitigating potential matrix effects.

Beyond the application to gout, the methodological framework established in this work possesses universality. Its universality was preliminarily validated by our group’s prior successful applications: the screening of thrombin inhibitors and SARS-CoV-2 main protease inhibitors from the of Xuebijing injection using analogous platform (Li et al., 2023; Han et al., 2024). This study has further demonstrated the efficacy of this approach by successfully screening XOD inhibitors from NF extract. This series of successful applications across distinct therapeutic targets (coagulation, antiviral and anti-gout) demonstrates that the proposed platform provides a reference-worthy paradigm. It offers a universal paradigm for the high-throughput discovery of bioactive metabolites from TCM targeting different diseases. The core strategy of “signal switching induced by enzyme catalyzed substrate hydrolysis” could be readily adapted to other enzyme targets, provided two key conditions were met: (1) the availability of a specific enzyme-substrate pair, and (2) the ability to conjugate the substrate into nanoparticles without compromising its recognition by the enzyme. To translate the identified inhibitors into practical applications, several follow-up studies are warranted. First, molecular docking and enzyme kinetics assays should be conducted to elucidate the binding modes and inhibition mechanisms of the four identified flavonoids. Second, *in vitro* cell-based assays using hepatic or intestinal cell lines could assess their cellular uric acid-lowering effects and potential cytotoxicity. Collectively, these investigations would not only validate the therapeutic potential of these natural compounds but also lay the groundwork for future development of TCM-derived anti-gout agents.

## Data Availability

The original contributions presented in the study are included in the article/Supplementary Material, further inquiries can be directed to the corresponding authors.
