# Supplementary material for: A universal fluorescent sensing platform based on “signal switching induced by enzyme catalyzed substrate hydrolysis” for high-throughput discovery of xanthine oxidase inhibitors from traditional Chinese medicine
Source: Front Pharmacol. 2026 Apr 21;17:1801989. doi: 10.3389/fphar.2026.1801989 (PMC13139129; doi:10.3389/fphar.2026.1801989)
Supplement: Supplementary file 1 [file Supplementaryfile1.docx]

***Supplementary Material***

**Methods Detail**

1. Integrated process steps of “synthesis---detection---separation---screening”:

A: Synthesis of 3-FCNA-Xan Nanoparticles (NPs)

(1)3-FCNA-Xan NPs were synthesized by a cross-linking method, and the synthesis conditions (including substrate concentration, stirring duration, stirring rate, and glutaraldehyde ratio) were optimized using the controlled variable method.

1. Slowly add 100 μL of tetrahydrofuran (THF) solution containing 10 mg/mL 3-FCNA to a vial containing 900 μL of 1 mM xanthine (Xan) solution, followed by slow addition of 9 μL of 25% glutaraldehyde.
2. Place the mixture on a magnetic stirrer and stir at 300 rpm for 60 min at room temperature under sealed and light-avoiding conditions. After the reaction, stand in the dark for 12 h to allow THF evaporation, then adjust the volume back to the original to obtain 3-FCNA-Xan NPs.
3. Store the synthesized 3-FCNA-Xan NPs in a refrigerator at 4°C away from light for later use.

B: Xanthine Oxidase (XOD) Detection Assay

1. Basic detection: Add PBS buffer to the wells of a black 96-well plate and pre-warm at 37°C for 5 min, add XOD and incubate for another 5 min; add 50 μL of 3-FCNA-Xan NPs solution, adjust the final reaction volume to 100 μL with PBS buffer, and immediately detect the fluorescence intensity using a multimode microplate reader (λex/λem = 542/690 nm); use allopurinol as a positive control.
2. Determination of enzyme activity curve: Add XOD at different concentrations (0.5, 5, 50, 100, 200, 300, 400, 500 and 600 U/L) to a black 96-well plate, shake slowly and incubate for 5 min, add 50 μL of 3-FCNA-Xan NPs solution, make up to 100 μL with PBS buffer, detect the fluorescence intensity with a multimode microplate reader (λex/λem = 542/690 nm), and draw the enzyme activity curve.

C: HPLC Fractionation + Screening Workflow

1. Preliminary screening of inhibitory activity of NF extract: Refer to the basic detection protocol in Section 2.2, after incubating with XOD for 5 min, add 10 μL of NF extract and continue incubating for 10 min, add 50 μL of 3-FCNA-Xan NPs solution, make up to 100 μL with PBS buffer, and detect the fluorescence intensity with a multimode microplate reader (λex/λem = 542/690 nm) to evaluate the inhibitory effect of the extract on XOD.
2. HPLC fractionation: Optimize mobile phase composition, detection wavelength, flow rate, column temperature and gradient elution conditions to construct the HPLC fingerprint of NF extract; collect fractions of NF extract every 1 min using an HPLC-fully automatic partial fraction collector (HPLC-FC).
3. Fractional activity screening and identification: Dry the collected fractions with nitrogen, then dissolve them in extraction solvent; evaluate the anti-XOD activity of each fraction using 3-FCNA-Xan NPs according to the above detection method; qualitatively analyze the fractions with significant anti-XOD activity by UHPLC-Q-TOF/MS, and determine the specific metabolites inhibiting XOD by comparing with standard samples.
4. Validation experiments: Determine the half-maximal inhibitory concentration (IC_50_) of active compounds by 3-FCNA-Xan NPs, validate the HPLC method for four compounds in terms of linearity, precision, stability, repeatability and matrix recovery, prepare a mixed reference standard containing each compound at the same concentration as in the extract, and compare its anti-XOD activity with that of the original whole extract.

**Supplementary Figures**


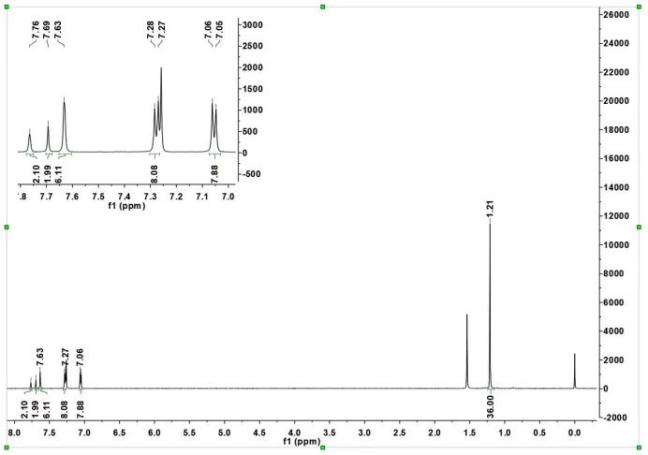


**Figure S1.** ^1^H NMR spectrum of 3-FCNA was provided by Ruixi Biotechnology Co., Ltd., Xi’an.

**Figure S2.** The MSMS fragment of 3-FCNA.





**Figure S3.** The hydrodynamic size of 3-FCNA determined by DLS.





**Figure S4.** The Zeta potential of 3-FCNA.





**Figure S5.** The Zeta potential of Xan.












**Figure S6.** Fluorescencehy, drodynamic diameter and zeta potential intensity of 3-FCNA-Xan NPs during 7 days and the stability of different groups.





**Figure S7.** Specific analysis of 3-FCNA-Xan NPs in the presence of different proteases or ions.





**Figure S8.** The effect of different reaction times on the detection of XOD in 3-FCNA-Xan NPs.





**Figure S9.** The time of optimization of XOD pre-incubation.





**Figure S10.** The time of optimization of inhibitor incubation time.





**Figure S11.** Use an UV-Vis spectrophotometer to detect the characteristic peaks of the reaction products between XOD and Xan.





**Figure S12.** Use an UV-Vis spectrophotometer to detect the characteristic peaks of the reaction products between XOD and 3-FCNA-Xan NPs, and observe the changes in the characteristic peaks of the products after adding XOD inhibitors.





**Figure S13.** Excluding the influence of NF extract on the fluorescence intensity of 3-FCNA-Xan NPs.

**

**

**Figure S14.** Effect of different mobile phases on separation of NF extract.





**Figure 15.** HPLC chromatograms of the NF extract at different wavelengths.





**Figure S16.** HPLC chromatograms of NF extract under different flow rate

conditions.





**Figure S17.** Effect of column temperature on the separation of NF extract.





**Figure S18.** HPLC chromatograms of NF extract under different extraction solvent.





**Figure S19.** The metabolites NF extract that have a high inhibitory rate on XOD.





**Figure S20.** HPLC chromatograms of NF extract and fraction 18 (F18).





**Figure S21.** HPLC chromatograms of NF extract and fraction 23 (F23).





**Figure S22.** HPLC chromatograms of NF extract and fraction 25 (F25).





**Figure S23.** HPLC chromatograms of NF extract and fraction 27 (F27).





**Figure S24.** HPLC chromatograms of NF extract and fraction 35 (F35).





**Figure S25.** HPLC chromatograms of NF extract and fraction 36 (F36).





**Figure S26.** HPLC chromatograms of NF extract and fraction 38 (F38).


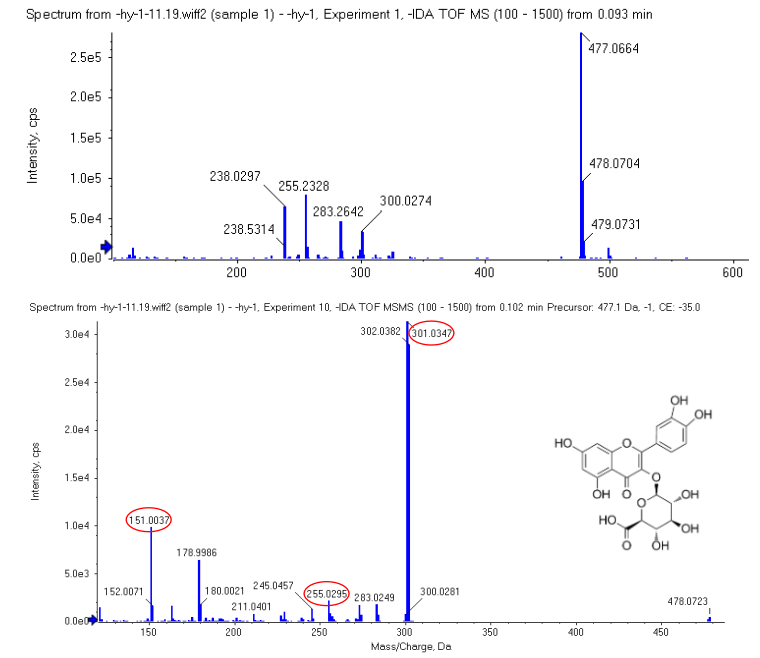


**Figure S27.** The MS and MS/MS fragments of fraction 8 (F8).


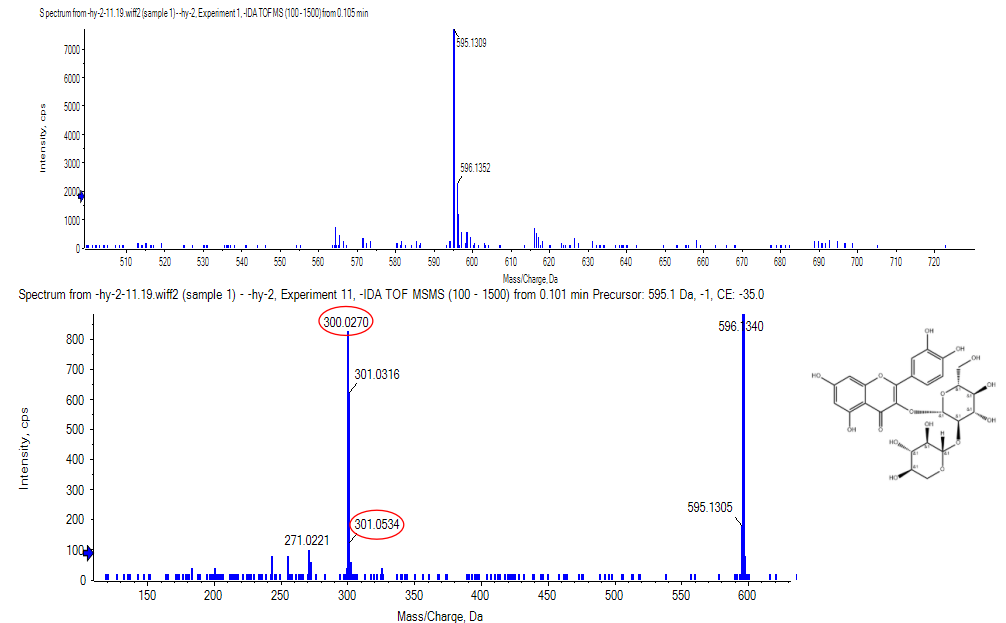


**Figure S28.** The MS and MS/MS fragments of fraction 18 (F18).


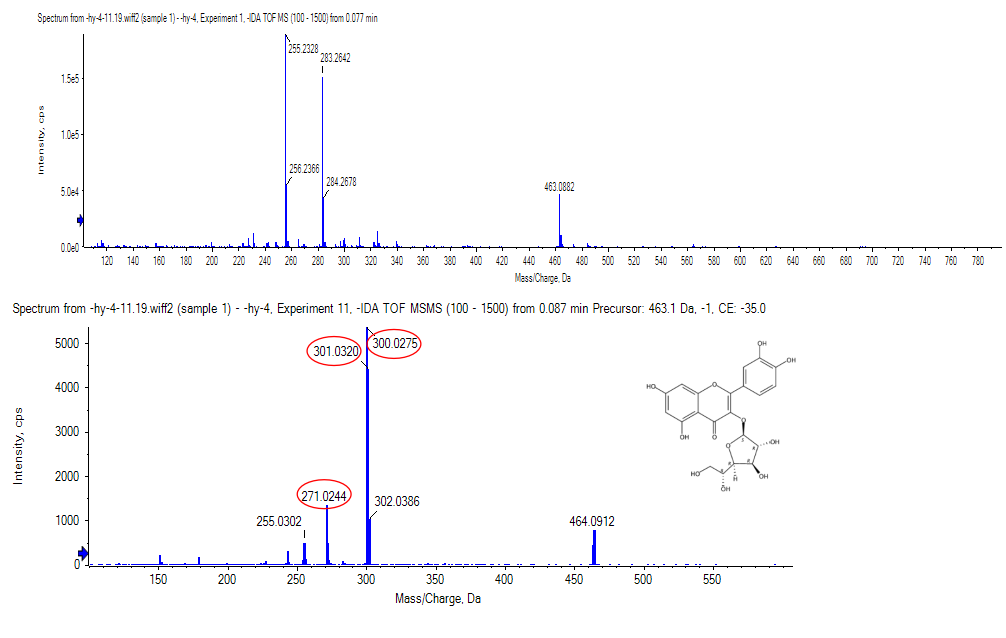


**Figure S29.** The MS and MS/MS fragments of fraction 25 (F25).


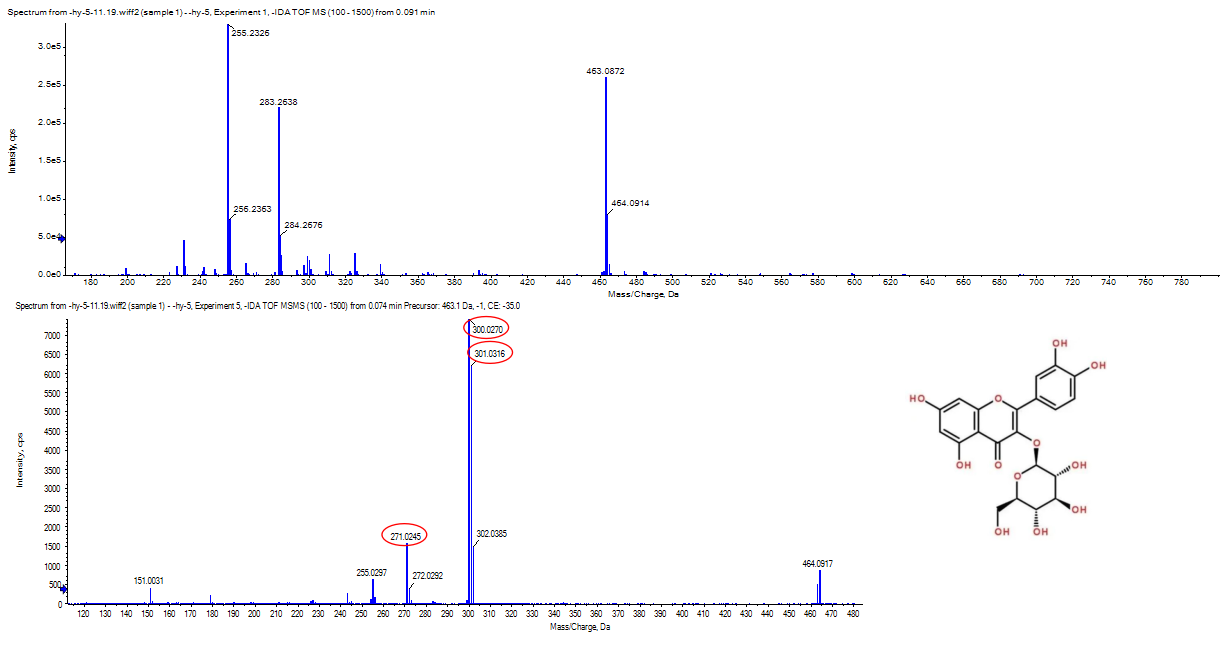


**Figure S30.** The MS and MS/MS fragments of fraction 26 (F26).


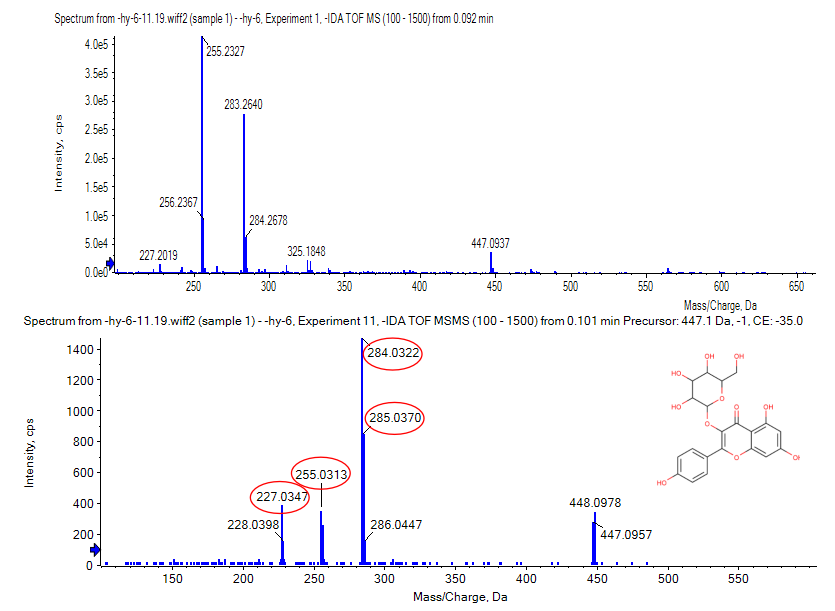


**Figure S31.** The MS and MS/MS fragments of fraction 35 (F35).


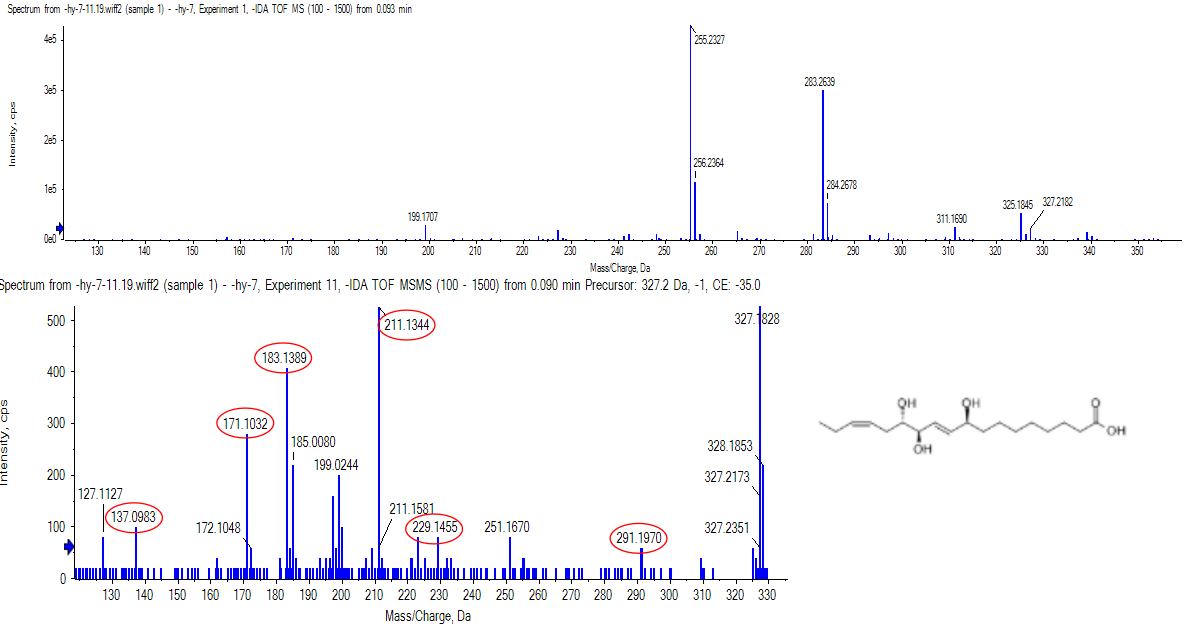


**Figure S32.** The MS and MS/MS fragments of fraction 36 (F36).


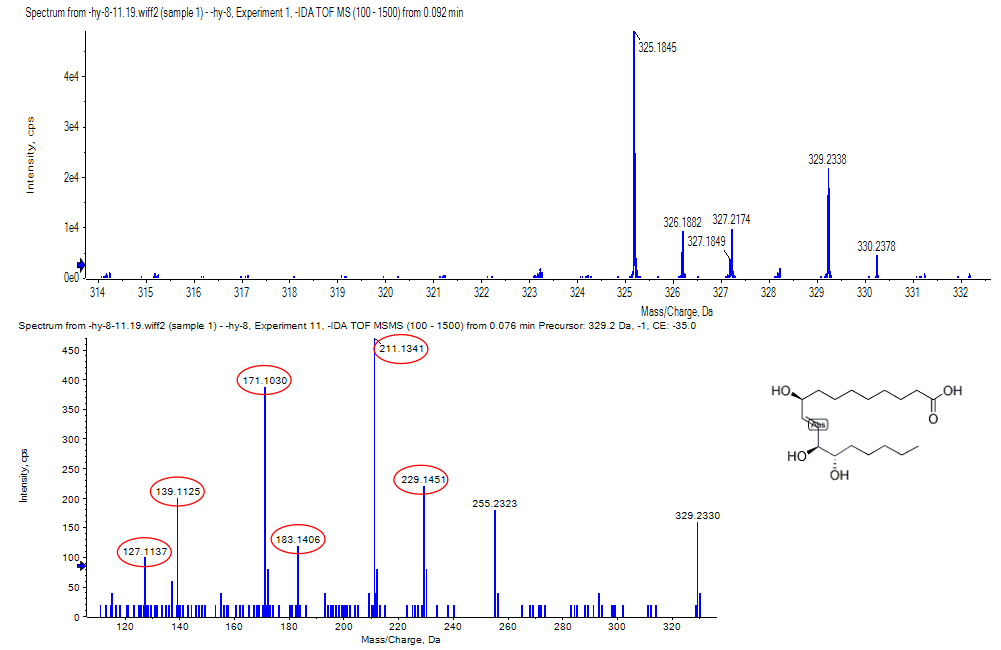


**Figure S33.** The MS and MS/MS fragments of fraction 38 (F38).





**Figure S34.** HPLC chromatograms of different samples.







**Figure S35.** A correlation standard curve between uric acid absorbance and XOD concentration was established using xanthine as a substrate.





**Figure S36.** Validate the inhibition rate measured by sensors using traditional methods.

**Supplementary Tables**

**Table S1.** Recovery of Allopurinol spiked into the assay matrix. (n=3)

| **Sample Name** | **Spiked (μM)** | **Measured (μM)** | **Average recovery (%)** | **Mean±SD** |
| --- | --- | --- | --- | --- |
| Allopurinol | 5 | 5.19 | 102.13 | 5.17±0.048 |
|  |  | 5.12 |  |  |
|  |  | 5.21 |  |  |
|  | 10 | 9.49 | 98.22 | 9.60±0.096 |
|  |  | 9.68 |  |  |
|  |  | 9.63 |  |  |
|  | 15 | 16.53 | 103.24 | 16.38±0.20 |
|  |  | 16.16 |  |  |
|  |  | 16.44 |  |  |

**Table S2.** Gradient elution procedure for HPLC.

| **Time(min)** | **A%** | **B%** |
| --- | --- | --- |
| 0 | 90 | 10 |
| 5 | 85 | 15 |
| 20 | 85 | 15 |
| 30 | 80 | 20 |
| 35 | 70 | 30 |
| 40 | 50 | 50 |
| 45 | 10 | 90 |

**Table S3.** Identification of high inhibitory metabolites against XOD in NF.

| **Fraction** | **formular** | **Identity** | **Classification** | **Found at Mass** | **ion** | **ppm** | **Fragmentation ions** |
| --- | --- | --- | --- | --- | --- | --- | --- |
| 8 | C_21_H_18_O_13_ | quercetin-3-0-glucuronide | flavonoids | 477.0675 | [M-H]^-^ | -2.2 | 301.0347、300.0281、151.0037 |
| 18 | C_26_H_28_O_16_ | quercetin-3-sambubioside | flavonoids | 595.1305 | [M-H]^-^ | 0.9 | 301.0316、300.0270 |
| 25 | C_21_H_20_O_12_ | quercetin-3-O-galactoside | flavonoids | 463.0882 | [M-H]^-^ | 0.2 | 301.0320、300.0275、271.0244 |
| 26 | C_21_H_20_O_12_ | quercetin 3-β-D-glucoside | flavonoids | 463.0882 | [M-H]^-^ | -0.4 | 301.0316、300.0270、271.0245 |
| 35 | C_21_H_20_O_11_ | kaempferol-3-0-glucoside | flavonoids | 447.0933 | [M-H]^-^ | 1.6 | 285.0370、284.0322、255.0313、227.0347 |
| 36 | C_18_H_32_O_5_ | 9,12,13-trihydroxyoctadecadienoic acid | fatty acid | 327.2177 | [M-H]^-^ | -0.6 | 291.1970、229.1455、211.1344、183.1389、171.1032、137.0983 |
| 38 | C_18_H_34_O_5_ | 9,12,3-rihydroxyotadecenoic acid | fatty acid | 329.2333 | [M-H]^-^ | 2.3 | 229.1451、211.1341、183.1406、171.1030、139.1125、127.1137 |

**Table S4.** Linear regression of the four metabolites.

| **Compounds** | **Regressive equation** | **Linear range (μg/mL)** | **R^2^** | **LOD (μg/mL)** | **LOQ (μg/mL)** |
| --- | --- | --- | --- | --- | --- |
| Quercetin-3-sambubioside | y = 12234.5x - 1981.14 | 1.0~100 | 0.9997 | 0.21 | 0.63 |
| Quercetin-3-O-galactoside | y = 16115.3x - 7819.6 | 2.5~250 | 0.9992 | 0.28 | 0.86 |
| Quercetin 3-β-D-glucoside | y = 14304.6x + 2403.54 | 1.0~100 | 0.9994 | 0.61 | 1.85 |
| Kaempferol-3-O-glucoside | y = 19870.6x + 238.67 | 0.1~10 | 0.9998 | 0.07 | 0.21 |

**Table S5.** Precision and stability of the four metabolites.

| **Compounds** | **Concentration (μg/mL)** | **Intraday** | | **Interday** | | **Stability** | |
| --- | --- | --- | --- | --- | --- | --- | --- |
|  |  | **RSD (%)** | **Accuracy (%)** | **RSD (%)** | **Accuracy (%)** | **RSD (%)** | **Accuracy (%)** |
| Quercetin-3-sambubioside | 10.0 | 0.71 | 100.20 | 1.27 | 100.17 | 0.87 | 99.92 |
|  | 50.0 | 0.33 | 100.81 | 0.90 | 100.17 | 0.49 | 100.69 |
|  | 100 | 0.75 | 100.77 | 0.90 | 100.60 | 0.90 | 100.75 |
| Quercetin-3-O-galactoside | 5.00 | 0.41 | 100.13 | 0.82 | 99.75 | 0.55 | 99.99 |
|  | 25.0 | 1.32 | 101.67 | 1.52 | 100.83 | 1.37 | 101.64 |
|  | 50.0 | 0.74 | 100.36 | 0.54 | 100.49 | 0.56 | 100.49 |
| Quercetin 3-β-D-glucoside | 10.0 | 1.85 | 100.69 | 1.47 | 99.36 | 2.26 | 100.39 |
|  | 50.0 | 2.10 | 100.89 | 1.02 | 99.93 | 2.55 | 100.74 |
|  | 100 | 1.75 | 100.78 | 1.16 | 98.62 | 2.07 | 100.93 |
| Kaempferol-3-O-glucoside | 1.00 | 1.06 | 103.42 | 1.49 | 103.80 | 0.96 | 103.03 |
|  | 5.00 | 0.29 | 95.67 | 0.68 | 95.97 | 0.69 | 96.08 |
|  | 10.0 | 0.93 | 99.22 | 1.27 | 99.57 | 1.08 | 99.45 |

**Table S6.** The repeatability of four metabolites.

| **Compounds** | **Concentration (μg/mL)** | **RSD (%)** |
| --- | --- | --- |
| Quercetin-3-sambubioside | 22.71 | 0.25 |
| Quercetin-3-O-galactoside | 7.84 | 0.12 |
| Quercetin 3-β-D-glucoside | 58.86 | 1.58 |
| Kaempferol-3-O-glucoside | 1.76 | 0.58 |

**Table S7.** Recoveries of spiked four metabolites.

| **Compounds** | **Original (μg)** | **Spiked (μg)** | **Found (μg)** | **Average recovery (%)** | **RSD (%)** |
| --- | --- | --- | --- | --- | --- |
| Quercetin-3-sambubioside | 22.71 | 18.17 | 40.71 | 99.59 | 0.27 |
|  |  | 22.71 | 45.49 | 100.16 | 0.50 |
|  |  | 27.25 | 49.66 | 99.40 | 0.53 |
| Quercetin-3-O-galactoside | 7.84 | 6.27 | 14.17 | 100.46 | 0.40 |
|  |  | 7.84 | 15.74 | 100.39 | 1.14 |
|  |  | 9.41 | 17.44 | 101.17 | 1.11 |
| Quercetin 3-β-D-glucoside | 58.86 | 47.09 | 109.64 | 103.48 | 0.84 |
|  |  | 58.86 | 120.54 | 102.39 | 1.50 |
|  |  | 70.63 | 134.40 | 103.78 | 0.47 |
| Kaempferol-3-O-glucoside | 1.76 | 1.41 | 3.16 | 99.89 | 0.74 |
|  |  | 1.76 | 3.57 | 101.45 | 0.44 |
|  |  | 2.11 | 3.87 | 99.86 | 0.42 |
